# Cellular immunotherapy for acute myeloid leukemia: from bench to bedside

**DOI:** 10.1093/immadv/ltag012

**Published:** 2026-07-08

**Authors:** Yu Wang, Jiaying Wu, Xiaobing Huang, Yi Xiao

**Affiliations:** Department of Hematology, Tongji Hospital, Tongji Medical College, Huazhong University of Science and Technology, Wuhan, Hubei P.R. China; Department of Hematology, Tongji Hospital, Tongji Medical College, Huazhong University of Science and Technology, Wuhan, Hubei P.R. China; Department of Hematology, Sichuan Academy of Medical Sciences & Sichuan Provincial People's Hospital, Chengdu, Sichuan P.R. China; Department of Hematology, Tongji Hospital, Tongji Medical College, Huazhong University of Science and Technology, Wuhan, Hubei P.R. China

**Keywords:** acute myeloid leukemia, chimeric antigen receptor T-cell therapy, chimeric antigen receptor natural killer cell therapy, chimeric antigen receptor macrophage therapy, tumor-infiltrating lymphocytes, cytokine-induced killer cells

## Abstract

Acute myeloid leukemia (AML) is a clonal malignant disease that arises from hematopoietic stem cells and is characterized by high genetic heterogeneity. AML has a poor prognosis, with a low 5-year survival rate, and is typically treated with chemotherapy. The management of AML, particularly for cases that are relapsed or refractory to conventional chemotherapy and transplantation, remains a formidable clinical challenge. This comprehensive review systematically evaluates the current landscape of cellular immunotherapeutic strategies for AML. We focus on the mechanisms of action, recent clinical progress, and persistent challenges associated with a range of adoptive cell therapies. These include natural killer cell-based infusions, and engineered approaches such as chimeric antigen receptor-T, CAR-NK, and CAR-macrophage therapies. Furthermore, we examine the roles of tumor-infiltrating lymphocytes and cytokine-induced killer cells. Our analysis synthesizes evidence from preclinical studies and clinical trials to assess the potential of these modalities in inducing durable antileukemic responses. We conclude that while cellular immunotherapies represent a transformative frontier in AML treatment, their broad application is currently constrained by target antigen heterogeneity, a complex tumor microenvironment, and significant toxicities. Finally, this review discusses strategic innovations necessary to overcome these barriers, highlighting directions for future research aimed at enhancing the efficacy and safety of these promising treatments for AML patients.

## Graphical Abstract

**Graphical Abstract:**
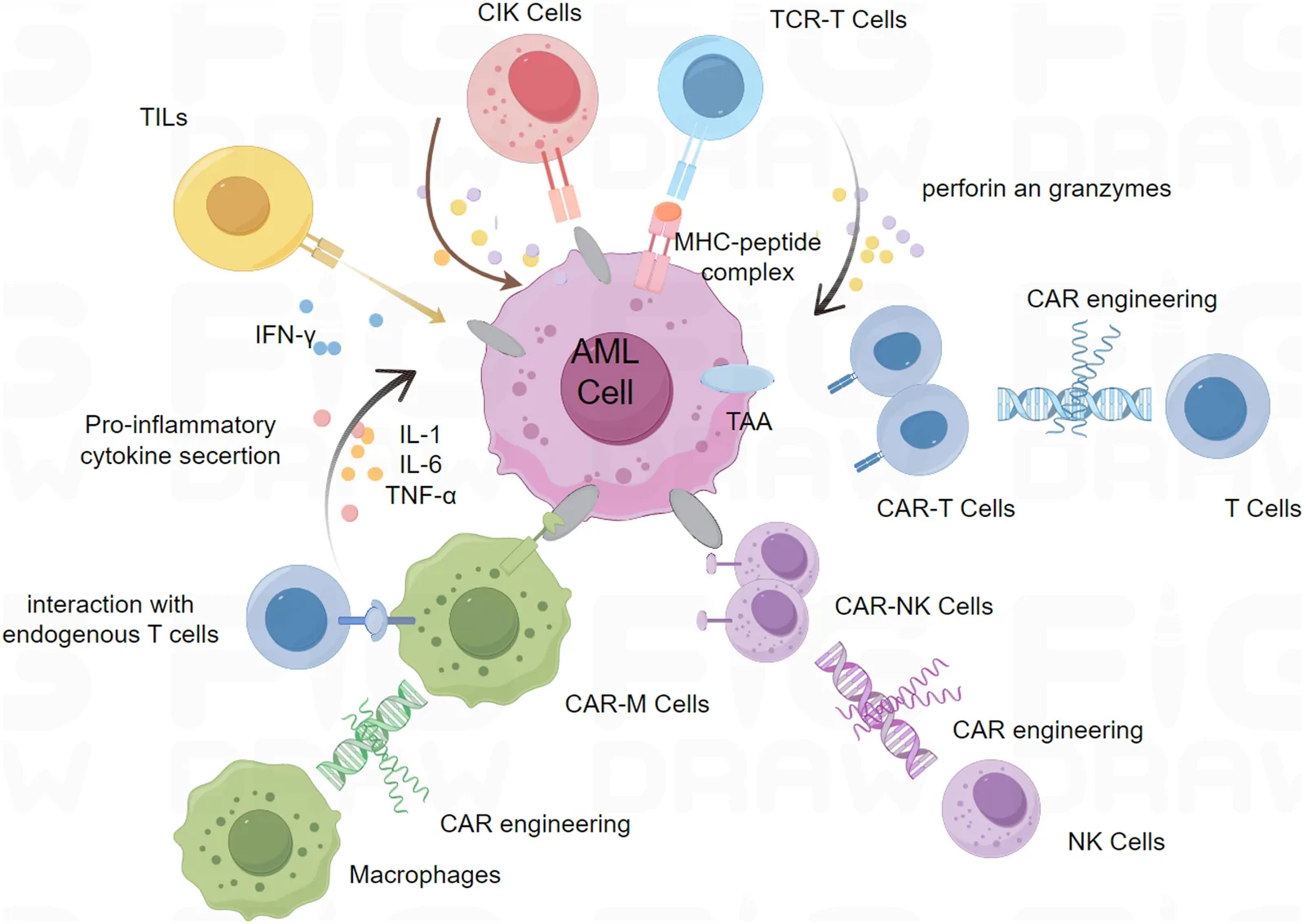

## Introduction

Acute myeloid leukemia (AML) is a genetically heterogeneous malignancy of hematopoietic stem/progenitor cells with high rates of primary resistance and relapse. Standard “3+7” induction achieves complete remission (CR) in approximately 60%–70% of patients, but responses are substantially lower in high-risk molecular subgroups. Among patients younger than 60 years, 35%–80% may relapse and 10%–40% develop primary resistance [1–3]. Allogeneic hematopoietic stem cell transplantation (allo-HSCT) improves outcomes for selected patients, although post-transplant relapse, graft-versus-host disease (GVHD), infection, and limited tolerance of salvage chemotherapy remain major problems [4]. These limitations have accelerated the development of adoptive cellular immunotherapies for relapsed/refractory (R/R) AML [5]. Current approaches include non-engineered natural killer (NK) cells, cytokine-induced killer (CIK) cells, and tumor-infiltrating lymphocytes (TILs), together with engineered chimeric antigen receptor (CAR)-T cells, CAR-NK cells, CAR-macrophages (CAR-Ms), and T-cell receptor-engineered T cells (TCR-T). These products use autologous or allogeneic immune cells that are expanded, activated, or genetically modified to improve recognition and elimination of AML cells. This review summarizes their biological basis, preclinical and clinical progress, and major barriers to clinical translation.

## Non-engineered cellular immunotherapy

AML progression reflects both clonal proliferation and escape from immune surveillance [6]. Within the bone marrow microenvironment, inhibitory receptors, dysregulated cytokines, and suppressive cellular interactions impair innate and adaptive immune function. Restoring antileukemic immunity therefore provides the rationale for non-engineered cellular therapies. This section summarizes the development of NK-cell, CIK-cell, and TIL-based approaches in AML.

### Natural killer cells

NK cells are pivotal effector cells of the innate immune system. Through the “missing-self” recognition mechanism and the detection of stress-induced ligands, NK cells can rapidly recognize and eliminate leukemic cells within minutes without prior antigen sensitization [6]. Specifically, when AML cells downregulate major histocompatibility complex class I (MHC-I) molecules to evade T-cell surveillance, inhibitory signaling mediated by killer-cell immunoglobulin-like receptors (KIRs) on NK cells is attenuated, thereby triggering NK cell cytotoxicity [7]. In addition, stress-induced ligands expressed on leukemic cells, including NKG2D ligands such as MICA/B and ULBP family proteins, can activate the NKG2D receptor on NK cells, leading to perforin/granzyme-mediated target-cell lysis as well as FasL- and TRAIL-dependent apoptotic pathways [8]. NK cells also secrete cytokines such as interferon-γ (IFN-γ) and tumor necrosis factor-α (TNF-α), which enhance macrophage- and T-cell-mediated antitumor immunity, promote dendritic cell maturation, and bridge innate and adaptive immune responses [6].

However, multiple immunosuppressive mechanisms within the AML microenvironment profoundly impair NK-cell function. Clinical studies have demonstrated that both the number and cytotoxic activity of peripheral blood NK cells are significantly reduced in patients with AML or myelodysplastic syndrome (MDS) compared with healthy individuals [9]. Moreover, the production of perforin, granzyme B, and IFN-γ is markedly diminished. Mechanistically, leukemic stem cells (LSCs) frequently lack expression of NKG2D ligands, thereby escaping NK-cell recognition. AML cells can also overexpress inhibitory ligands such as programmed death-ligand 1 (PD-L1), which suppress NK-cell activation through interaction with PD-1 on NK cells [6]. In parallel, transforming growth factor-β (TGF-β) derived from bone marrow stromal cells inhibits mammalian target of rapamycin complex 1 (mTORC1) signaling and mitochondrial oxidative phosphorylation in NK cells, ultimately impairing their proliferation and cytotoxic capacity [10].

Early clinical experience with autologous NK cells showed safety but limited efficacy. In a phase I/II study, IL-2-expanded autologous NK cells increased transiently after infusion, yet the single patient with AML achieved only a minor reduction in bone marrow blasts and progressed within 30 days [11]. Intrinsic dysfunction of patient-derived NK cells may partly explain this result.

Allogeneic NK cells showed stronger activity. In a phase I trial, 19 patients with AML received haploidentical donor NK cells after lymphodepletion and IL-2 support [12]. Donor cells expanded *in vivo*, and five patients achieved CR; four maintained remission for a median of 9 months. One patient underwent allo-HSCT and remained disease-free for more than 24 months. These findings established the feasibility of donor NK-cell therapy outside a transplant platform.

The graft-versus-leukemia effect of alloreactive NK cells was demonstrated in haploidentical allo-HSCT. Ruggeri *et al*. [13] found that KIR-ligand incompatibility in the graft-versus-host direction was associated with a 5-year AML relapse probability of 0%, compared with 75% in compatible transplants, and improved event-free survival. Acute GVHD and graft rejection were also reduced. These observations provided a biological framework for selecting donors and developing alloreactive NK-cell products.

Cytokine-induced memory-like (CIML) NK cells are generated by brief IL-12, IL-15, and IL-18 preactivation, producing epigenetic and metabolic changes that enhance secondary responses and persistence [14, 15]. In a phase I trial of six patients with post-transplant myeloid malignancies, donor-derived CIML NK cells expanded 10–50-fold, persisted for several months, and produced mainly fever and pancytopenia [14]. Subsequent combination with the IL-15 superagonist N-803 and ipilimumab increased early proliferation but accelerated donor-cell rejection, indicating that cytokine and checkpoint combinations require careful optimization [15].

Umbilical cord blood provides another potentially off-the-shelf NK-cell source. In a phase II study of 51 older patients with AML, consolidation chemotherapy combined with cord blood infusion produced 2-year OS and PFS rates of 76.9% and 62.8%, respectively, without acute GVHD or treatment-related mortality [16]. Single-cell analysis suggested enhanced immune activation and antisenescence programs in cord-blood-derived immune cells.

Despite the promising therapeutic potential of NK-cell-based immunotherapy in AML, several major clinical challenges remain. Large-scale manufacturing is hindered by limited expansion efficiency and substantial phenotypic heterogeneity. In addition, the terminal differentiation phenotype of umbilical cord blood-derived NK cells may compromise their *in vivo* persistence. Prolonged ex vivo stimulation can also induce excessive expression of apoptotic ligands such as FasL, leading to autocrine or paracrine apoptosis of NK cells themselves. Collectively, these limitations have prompted increasing interest in genetic engineering approaches aimed at enhancing NK-cell proliferation, persistence, and antileukemic activity.

### Cytokine-induced killer cells

Cytokine-induced killer (CIK) cells are a heterogeneous population of T lymphocytes with NK cell-like activity. They are generated from peripheral blood or umbilical cord blood mononuclear cells through ex vivo stimulation with anti-CD3 antibodies and cytokines such as interleukin-2 (IL-2) and IFN-γ. CIK cells possess both the antigen-specific cytotoxic capacity of T cells (CD3^+^CD56^−^) and the non-MHC-restricted cytotoxicity characteristic of NK-like cells (CD3^+^CD56^+^). CIK cells exert antileukemic effects against AML through multiple mechanisms. First, they release perforin to form pores in the target-cell membrane, allowing granzyme B entry and subsequent activation of the caspase cascade to induce apoptosis. Second, surface Fas ligand (FasL) or TNF-related apoptosis-inducing ligand (TRAIL) can bind to death receptors (Fas or DR4/5) on AML cells, thereby triggering apoptotic signaling pathways. Third, recognition of stress-induced ligands, including MICA/B and ULBP family proteins, through the NKG2D receptor further enhances targeted cytotoxicity [17]. Because CIK-cell-mediated killing is independent of MHC-restricted antigen presentation, these cells can effectively eliminate AML cells with low MHC expression, including LSCs, thereby overcoming tumor immune evasion. In addition, CIK cells secrete proinflammatory cytokines such as IFN-γ and TNF-α, which activate macrophages and dendritic cells and contribute to the establishment of a more favorable antitumor immune microenvironment.

CIK cells expand rapidly and have relatively low alloreactivity, which may reduce GVHD risk [18]. They inhibit AML growth in xenograft models [19]. Cord-blood-derived CIK cells may offer greater proliferative capacity, lower immunogenicity, and resistance to chemotherapy-induced apoptosis than peripheral-blood-derived products.

Clinical evidence remains heterogeneous. In 36 patients with relapsed hematologic malignancies after allo-HSCT, CIK-cell infusion produced a 53% CR rate, a 6-month OS rate of 77%, and acute GVHD in 25% [20]. Registry analyses and dendritic cell-CIK combinations have reported survival benefits in several malignancies, generally without severe treatment-related toxicity [21, 22], although AML-specific prospective evidence is limited.

CIK cells are currently better supported as adjunctive therapy. Standardized manufacturing and large AML-specific trials are required to define their efficacy and optimal combinations.

### Tumor-infiltrating lymphocytes

Tumor-infiltrating lymphocytes (TILs) are a heterogeneous population of lymphocytes residing within tumor tissues. In AML, TILs are predominantly enriched in the bone marrow microenvironment. A major advantage of TILs lies in their intrinsic expression of receptors targeting tumor-associated antigens, such as Wilms tumor 1 (WT1) and preferentially expressed antigen in melanoma (PRAME), enabling recognition of a broader antigen repertoire and thereby overcoming tumor heterogeneity. Importantly, TIL cultures are heterogeneous and contain only a subset of tumor-reactive T cells capable of recognizing AML-associated antigens. This characteristic provides a strong rationale for the application of TIL-based immunotherapy in AML.

In AML, TILs are primarily localized within the bone marrow niche. Wei *et al*. [23] conducted a systematic analysis of bone marrow samples from AML patients and demonstrated that 33%–50% of patients harbored TIL populations capable of secreting IFN-γ. These cells could be successfully expanded ex vivo and exhibited measurable antitumor activity. Teo *et al*. [24] further demonstrated that TILs isolated from the bone marrow of AML patients could be reactivated using a simulated infection protocol (SIP) and subsequently converted into T cells with a central memory phenotype (CD45RA^+^), thereby enhancing their persistence and antitumor potential.

However, not all cells within expanded TIL cultures exhibit tumor reactivity. Rather, TIL cultures serve as a source from which tumor-reactive T-cell clones can be identified, selected, and expanded ex vivo to enrich populations with potent antitumor activity. Moreover, TILs derived from AML patients frequently display terminal differentiation or exhaustion phenotypes, such as the CD69^+^/Ly108^−^ phenotype, which may impair their proliferative capacity and cytotoxic activity following ex vivo expansion, ultimately limiting their therapeutic efficacy in AML. Persistent antigenic stimulation within the AML microenvironment further exacerbates TIL dysfunction, leading to loss of effector function, reduced proliferative potential, and upregulation of inhibitory receptors including programmed cell death protein 1 (PD-1), T-cell immunoglobulin and mucin-domain containing-3 (TIM-3), and lymphocyte activation gene-3 (LAG-3) [25]. In addition, regulatory T cells (Tregs) within the microenvironment further suppress TIL function through competitive consumption of IL-2 and secretion of immunosuppressive cytokines [25]. The absence of standardized protocols for TIL isolation, selection, expansion, and reinfusion also contributes to variability and limited reproducibility in clinical outcomes.

Accordingly, future development of TIL-based therapy in AML should focus on several key areas: validating long-term clinical efficacy through large-scale clinical trials; developing strategies to overcome the immunosuppressive bone marrow microenvironment, such as combination therapy with immune checkpoint inhibitors; optimizing TIL manufacturing procedures to improve enrichment of tumor-reactive clones; and exploring combinational approaches with chemotherapy, targeted agents, or other immunotherapeutic modalities.

### Limitations of non-engineered therapies and the need for engineering

Non-engineered NK cells, CIK cells, and TILs show antileukemic potential but share limitations in persistence, functional fitness, manufacturing consistency, and monotherapy efficacy. These constraints support the use of CAR and TCR engineering to improve antigen specificity, cellular durability, and activity within the AML microenvironment.

## Principle of action and target of CAR technology

CAR is a genetically engineered construct designed to recognize specific antigens and activate effector cells. It comprises four essential structural domains: (i) an extracellular antigen-binding domain consisting of a single-chain variable fragment (scFv) that recognizes target antigens; (ii) a hinge region linking the extracellular and transmembrane domains; (iii) a transmembrane domain that anchors the receptor to the cell membrane; and (iv) an intracellular signaling domain containing co-stimulatory molecules (such as CD28 and 4-1BB) and a T-cell activation domain (CD3ζ), which enhance cell activation and persistence [26]. CAR T-cell therapy has substantially advanced the treatment of hematological malignancies, particularly by improving the prognosis of patients with B-cell and plasma cell tumors [27, 28]. It has also emerged as a vital treatment option for R/R AML. Current research on AML treatment focuses on antigens, such as CD33, CD123, and C-type lectin-like molecule-1 (CLL-1) (Table 1). Beyond CAR T cells, novel therapies based on CAR technology, including CAR-NK and CAR-macrophages (CAR-Ms), are also emerging in tumor immunotherapy.

**Table 1:** Possible antigen targets for CAR T cells in patients with AML.

Antigen	% positive AML blasts	Expression on LSCs	Expression on healthy hematopoietic cells
CD33[29]	90%	+	Myeloid progenitor cells, unipotent colony-forming cells, maturing granulocytes, and monocytes
CD123[30, 31]	70%–80%	+	Low level/no expression on HSCs monocytes, plasmoid dendritic cells basophils
CLL-1[32]	78%–92%	+	granulocytes, monocytes. and their progenitors
CD7[33]	20%–30%	—	T lymphocytes, NK cells, and some myeloid cells.
CD38[34]	60%–70%	—	B lymphocytes, plasma cells, red blood cells, myeloid cells, and platelets.
FLT3[35]	54%–92%	+	HSCs, myeloid progenitor cells
CD47[36]	25%	—	B lymphocytes, plasma cells, red blood cells, myeloid cells, and platelets.
CD44v6[37]	80%	—	Monocytes, a small number of lymphocytes, and stromal cells.
Lewis Y (LeY)[38]	70%–90%	—	Lewis Y is expressed at low levels in the hematopoietic system
FRβ[39]	70%	—	FRβ is expressed at low levels in monocytes and macrophages

### CAR T-cell therapy

Building on the substantial achievements of CAR T-cell therapy in B-cell malignancies, the scientific community is now vigorously investigating its applicability in AML. Despite the absence of a CAR T-cell product currently approved specifically for AML, multiple clinical studies have revealed its promising therapeutic potential. Unlike B-cell malignancies that possess a universal target such as CD19, AML lacks a comparable antigen. Consequently, CAR T cells have been engineered to target a variety of antigens. The primary antigens currently under clinical investigation include CD33, CD123, CD7, CLL-1, CD47, CD19, CD38, and NKG2D.

#### CAR T cells targeting CD33

CD33 is expressed on AML blasts in approximately 85%–90% of patients but is also present on normal myeloid progenitors and mature myeloid cells [40, 41]. CD33 CAR T cells eliminate AML cells *in vitro* and reduce leukemia burden in mouse models [42]. In a study of 12 patients who relapsed after allo-HSCT, five achieved CR, but all developed myelosuppression and 10 experienced grade 1–2 CRS; six died from infection or disease progression [3]. Five responders proceeded to a second allo-HSCT and survived during the reported follow-up, supporting CAR T cells as a potential bridge to transplantation. Newer CD33 CAR constructs have improved expansion, viability, and cytotoxicity in preclinical studies [43]. Combination or multitarget strategies and epigenetic agents are also being evaluated to reduce antigen escape [44] (Table 2). The principal unresolved problem is sustained on-target injury to normal myelopoiesis.

**Table 2:** Registered clinical trials using CD33-chimeric antigen receptor T-cell for acute myeloid leukemia.

NCT/Registry number	Target antigen	Study title	Conditions	Sample size
NCT06762132	CD33	CD33 CAR-T for R/R AML	AML; R/R AML	27
NCT06420063	CD33/CD123	Sequential CD33/CD123 CAR-T for R/R AML	AML;R/R AML	85
NCT06326021	CD33 (FL-33)	Optimized FL-33 CAR-T for R/R AML	R/R AML	27
NCT05995041	CLL-1/CD33/CD38/CD123	Universal CAR-T for AML	R/R AML	30
NCT05984199	CD33 (VCAR33)	Donor-derived anti-CD33 CAR-T (VCAR33) post-allo-HSCT	R/R AML	38
NCT05672147	CD33	CD33 CAR-T with lymphodepletion for R/R AML	R/R AML	27
NCT05105152	CD33 (SC-DARIC33)	SC-DARIC33 in pediatric/young adult R/R CD33+ AML	R/R AML	18
NCT04835519	CD33	Functionally enhanced CD33 CAR-T for R/R AML	AML; R/R AML	5
NCT03126864	CD33	Autologous lentiviral CD33 CAR-T for R/R CD33+ AML	Hematopoietic/Lymphoid Cancer; AML; R/R AML	11
ChiCTR1800018669	CD33/CD123	Dual CD33-CD123 CAR-T for R/R AML	AML; R/R AML	10
ChiCTR1800017290	CD33	CD33 CAR-T for R/R AML	AML; R/R AML	30

#### CAR-T cells targeting CD123

CD123, the α-subunit of the interleukin-3 receptor (IL-3R), is a member of the common β (βC) cytokine receptor family. It primarily mediates IL-3 signaling, thereby regulating the proliferation, differentiation, and survival of hematopoietic and immune cells. Unlike CD33, CD123 is expressed at relatively low levels in the normal hematopoietic system but is frequently overexpressed in hematologic malignancies. Overactivation of CD123 has been implicated in tumor progression and relapse. In AML, high CD123 expression is observed in approximately 70%–80% of patients [31, 45], positioning CD123 as a promising therapeutic target [46]. An *in vivo* study [47], compared the efficacy and safety of genetically engineered immune cells expressing anti-CD33 or anti-CD123 CARs for AML. Both modified T-cell types effectively reduced the leukemia burden *in vivo*. However, only anti-CD123 CARs demonstrated lower cytotoxicity against normal hematopoietic stem/progenitor cells, highlighting their ability to balance antitumor efficacy with reduced adverse myelosuppressive effects. Consequently, CD123 has emerged as a highly attractive immune target for AML therapies.

In one patient with FUS-ERG+ R/R AML, anti-CD123 CAR T therapy was incorporated into a haploidentical transplant conditioning regimen. Within 2 weeks post-infusion, abnormal cells in the Bone marrow markedly decreased, accompanied by a significant increase in CAR copy number. The patient achieved complete donor chimerism, incomplete blood count recovery, and CR, suggesting that anti-CD123 CAR T therapy can effectively eliminate chemoresistant cells in FUS-ERG+ AML without impairing donor chimerism or Bone marrow engraftment [48]. However, the patient subsequently failed to achieve transfusion independence for platelets and red blood cells and died from severe acute GVDH, multiorgan dysfunction, and infection. Although several studies confirm the therapeutic efficacy of anti-CD123 CAR T therapy in R/R AML, hematologic toxicity remains a major challenge. To address the safety limitations of conventional CD123 CAR-T cells, alternative platforms with built-in controllability have been actively pursued.

One option for more controllable and targeted therapeutic strategy is to use a rapidly switchable universal CAR T (UniCAR) system, which consists of two separate components: (i) autologous T cells genetically engineered to express a UniCAR that recognizes a small peptide epitope (E5B9 tag) rather than a tumor antigen itself and (ii) a soluble targeting module (TM) comprising an antigen-binding domain (e.g. an scFv against CD123) fused to the E5B9 peptide epitope [49]. Under physiologic conditions, UniCAR-T cells remain inactive because the UniCAR extracellular domain does not recognize any surface protein on tumor cells. Only in the presence of the TM does the UniCAR-T cell become cross-linked to the target cell, resulting in CAR-T activation and tumor cell killing (ON switch). Upon withdrawal of the TM, CAR-T activity is rapidly terminated (OFF switch) due to the short pharmacokinetic half-life of the TM (approximately 0.45 hours preclinically), providing a built-in safety mechanism [49].

Building on this platform, a novel CD123-specific TM variant (TM123-4-1BBL) was engineered to enhance UniCAR-T cell function within the leukemia microenvironment. This variant incorporates a trimeric single-chain 4-1BBL into the targeting module, which transiently delivers co-stimulatory signals to UniCAR-T cells in *trans* at the tumor site [50]. Experimental data revealed that TM123-4-1BBL-directed UniCAR-T cells efficiently eliminated CD123-positive AML cells both *in vitro* and in cell line-derived xenograft (CDX) *in vivo* models. In addition to its primary targeting function, TM123-4-1BBL enhanced UniCAR-T cell expansion, persistence, and phenotypic modulation compared with standard TM123 [50].

In a first-in-human phase Ia trial of UniCAR-T-CD123, three patients with R/R AML received a single UniCAR T-cell infusion followed by continuous TM123 administration [49]. One achieved partial remission and two achieved CR with incomplete hematologic recovery. No dose-limiting toxicity occurred, and two patients developed only grade 1 CRS. Myelosuppression recovered after TM123 withdrawal, while UniCAR T cells remained detectable for at least 6 months. These findings provide early evidence that a rapidly controllable platform may improve safety.

Beyond the UniCAR platform, other strategies to enhance CD123 CAR-T efficacy include optimizing the CAR construct itself. For instance, using a humanized CSL362 scFv and integrating a CD28-OX40-CD3ζ composite signaling domain can reduce immunogenicity while enhancing T-cell proliferation and persistence. Compared with a single co-stimulatory domain such as 4-1BB, a composite CD28-OX40 domain may better balance activation and cytokine release toxicity [51]. In addition, CD123 expression in leukemia cells can be upregulated by azacitidine pretreatment. In murine leukemia models, azacitidine increased the proportion of CTLA-4-negative anti-CD123 CAR-T cells, which exhibited superior cytotoxicity against AML cells, higher TNFα secretion, and enhanced downstream phosphorylation of T-cell activation molecules. These findings suggest that azacitidine increases AML immunogenicity, thereby improving recognition and elimination by highly efficient CTLA-4-negative anti-CD123 CAR-T cells [52]. Dual- or multitarget CAR-T cells are also under development to increase tumor targeting, overcome heterogeneity, and reduce immune escape. Ongoing clinical trials of anti-CD123 CAR-T cells are listed in Table 3.

**Table 3:** Registered clinical trials using CD123-chimeric antigen receptor T-cell for acute myeloid leukemia.

NCT/Registry number	Target antigen	Condition	Sample size	Phase	Status	Intervention
NCT06420063	CD33/CD123	R/R AML	85	Phase 1/2	Recruiting	CD123/CD33 CAR-T
NCT06125652	Tim-3/CD123	R/R AML	20	Phase 1/2	Recruiting	Anti-Tim-3/CD123 CAR T
NCT05995041	CLL-1/CD33/CD38/CD123	AML	30	Phase 1	Recruiting	Multi-target universal CAR-T
NCT04318678	CD123	AML/MDS, B-ALL, T-ALL, BPDCN	32	Phase 1	Recruiting	CD123-CAR T + lymphodepletion
NCT03672851	CD123	Acute leukemia	2	Phase 1	Terminated	Anti-CD123 CAR T
ChiCTR1800018669	CD33/CD123	R/R AML	10	Phase I	Recruiting	Dual CD33-CD123 CAR T
ChiCTR1800017843	CD123	CD123-positive R/R myeloid leukemia	50	—	Recruiting	Anti-CD123 CAR T

B/T-ALL, B/T-cell acute lymphoblastic leukemia; BPDCN, blastic plasmacytoid dendritic cell neoplasm.

#### CAR T cells targeting CLL-1

CLL-1 is highly expressed on AML cells and leukemia stem cells, with more restricted expression in normal hematopoiesis than CD33 [32, 53, 54]. This pattern supports selective targeting, although expression on normal granulocytic compartments can still cause clinically relevant cytopenias.

CLL-1 CAR T cells show potent activity against AML cell lines, primary blasts, and disseminated xenograft models while preserving normal progenitor colony formation in several preclinical studies [55, 56]. These findings suggested a wider therapeutic window than that of CD33-directed therapy.

In a study of 10 adults with R/R AML, CLL-1 CAR T-cell therapy produced CR with incomplete hematologic recovery in seven patients [57]. All patients developed CRS and severe pancytopenia; two died from infection during agranulocytosis. Because CLL-1 is expressed on normal granulocytes, allo-HSCT may be required to restore hematopoiesis after response. In a separate analysis, bone marrow CR rates were 65.0% in patients with extramedullary disease and 81.5% in those without it, with no significant difference in CAR T-cell expansion or cytokine release [58]. These results support activity in both marrow and extramedullary AML, but prolonged myelosuppression and infection remain major limitations. Larger studies should define durability, supportive care, and the role of dual-target or combination strategies (Table 4).

**Table 4:** Registered clinical trials using CCL-1 chimeric antigen receptor T-cell for acute myeloid leukemia.

NCT/Registry number	Target antigen	Condition	Sample size	Phase	Status	Intervention
NCT06917105	CD33/CD123/CLL-1	Myeloid malignancies	45	Phase 1/2	Not yet recruiting	CD33/CD123/CLL-1 CAR T cells
NCT06307054	CLL-1	R/R AML	24	Phase 1	Recruiting	Anti-CLL-1 CAR-NK cells
NCT06128044	CLL-1	R/R AML	12	Phase 1	Terminated	CB-012 (CRISPR-edited allogeneic anti-CLL-1 CAR T)
NCT05995041	CLL-1/CD33/CD38/CD123	R/R AML	30	Phase 1	Recruiting	Universal CAR-T cells
NCT04219163	CLL-1	R/R AML	18	Phase 1	Recruiting	CLL-1.CAR-T cells
NCT04010877	CLL-1/CD33/CD123	R/R AML	10	Phase 1/2	Unknown status	Multi-target CAR-T cells
ChiCTR2200061646	CLL-1/CLL-1+CD33/CD33/ADGRE2	R/R AML	20	—	Recruiting	CAR T cells
ChiCTR2200060930	CLL-1	R/R AML	10	—	Recruiting	Autogenous CLL-1 CAR T cells
ChiCTR2200057594	CLL-1	R/R AML	68	—	Recruiting	Anti-CLL1 CAR T
ChiCTR2100046261	CLL-1	R/R AML	10	—	Unknown	Autologous CLL-1 CAR T

#### CAR T cells targeting CD7

CD7 is expressed on T cells and NK cells and is aberrantly present on AML blasts in approximately 20%–30% of patients [33, 59, 60]. CD7-positive AML is associated with chemoresistance and poor prognosis [61]. Its expression on normal lymphocytes creates a risk of CAR T-cell fratricide and prolonged immune deficiency.

CD7 positivity may coexist with DNMT3A mutations and an immature CD34^+^CD117^+^CD123^+^ phenotype. DNMT3A mutations are common early events in AML and clonal hematopoiesis [62, 63], and their combination with CD7 expression has been associated with lower CR rates and shorter survival [64].

These biological features support CD7 as a target in a molecularly and immunophenotypically adverse subgroup, although they do not establish CD7 as a leukemia-specific antigen.

CD7 CAR T cells can directly eliminate CD7-positive AML cells and may enhance host immunity through IFN-γ secretion [65]. Strategies to prevent fratricide include CD7 knockout, intracellular retention, or selection of naturally CD7-negative CAR T cells.

Naturally selected CD7 CAR T cells developed by Lu *et al*. [66] lost surface CD7 after transduction without gene editing and efficiently killed CD7-positive AML cell lines and primary R/R AML blasts *in vitro* and in xenograft models.

RD13-01a is a healthy-donor-derived CD7 CAR T product engineered to reduce fratricide, GVHD, and allogeneic rejection [67]. In a phase I trial, 9 of 11 evaluable patients responded and seven achieved CR. No severe CRS, neurotoxicity, GVHD, or dose-limiting toxicity occurred. However, cytomegalovirus or Epstein-Barr virus reactivation was observed, including one fatal EBV-associated lymphoma, emphasizing the need for intensive viral monitoring.

A phase I trial evaluated nanobody-based naturally selected CD7 CAR T cells in 10 patients with CD7-positive R/R AML [68]. Seven achieved CR, and three responders underwent allo-HSCT. Three of four patients who did not undergo transplantation relapsed within 90 days, while CD7 loss was detected in nonresponders and relapsed cases. CRS was usually mild and no neurotoxicity occurred. These findings not only support the use of CD7 CAR T cells as a bridge to allo-HSCT but also identify antigen loss as a major resistance mechanism [68, 69].

A sequential strategy combining CD7 CAR T cells with haploidentical HSCT without conventional myeloablation or GVHD prophylaxis was studied in 10 patients with CD7-positive leukemia or lymphoma [70]. All achieved CR with incomplete hematologic recovery before transplantation. At a median follow-up of 15.1 months, six remained in MRD-negative CR; the estimated 1-year OS and disease-free survival rates were 68% and 54%, respectively. Two fatal septic shock events and grade 4 pancytopenia in all patients show that profound aplasia and infection remain central safety concerns. Careful patient selection, prophylaxis, and supportive care are required. Anti-CD7 CAR T Cell trials are summarized in Table 5.

**Table 5:** Registered clinical trials using CD7 chimeric antigen receptor T-cell for acute myeloid leukemia.

NCT/Registry number	Target antigen	Condition	Sample size	Phase	Status	Intervention
NCT06934382	CD7	R/R T-ALL/T-LBL	33	Phase 1	Recruiting	Allogeneic anti-CD7 CAR T cells (BEAM-201)
NCT06925464	CD7	R/R mature T-cell lymphomas (PTCL/CTCL)	36	Phase 1/2	Recruiting	Autologous/new donor CD7 CAR T cells
NCT06787560	CD7	Bone marrow failure syndrome	20	Early Phase 1	Recruiting	CD7 CAR T cells + allo-HSCT
NCT06769191	CD7	Schimke immuno-osseous dysplasia	20	Early Phase 1	Recruiting	CD7 CAR T cells + allo-HSCT + kidney transplantation
NCT06720324	CD7	R/R CD7+ hematologic malignancies	80	Phase 1/2	Recruiting	Fludarabine + cyclophosphamide + CD7-specific CAR T cells
NCT06633328	CD7	Severe aplastic anemia	30	Early Phase 1	Recruiting	Donor-derived CD7 CAR T cells + allo-HSCT
NCT06622694	CD7	R/R severe aplastic anemia	15	Phase 1	Not yet recruiting	Universal CD7 CAR T cells (RD13-02)
NCT06514794	CD7	R/R T-ALL/lymphoblastic lymphoma	125	Phase 2	Recruiting	Anti-CD7 allogeneic CAR T cells (WU-CAR-T-007)
NCT06585345	CD7	R/R acute leukemia	200	Phase 1/2	Recruiting	CD7 CAR T cells
NCT06420076	CD7/CD5	R/R T-ALL, ETP-ALL, TLBL	60	Phase 1/2	Recruiting	Anti-CD7/CD5 CAR T cells
NCT06316427	CD7	R/R T-cell malignancies	80	Phase 1/2	Recruiting	Autologous/donor-derived CD7 CAR T cells
NCT06136364	CD7	R/R T-LBL/ALL	9	Phase 1	Recruiting	SENL101 autologous CAR T cells
NCT06064903	CD7	R/R CD7+ T-ALL/lymphoma (pediatric)	26	Phase 1/2	Recruiting	CD7-CART01
NCT05995028	CD7	T-ALL, T-lymphoblastic lymphoma, AML, NK cell lymphoma	30	Phase 1	Recruiting	Universal CD7-specific CAR T cells (4SCAR7U)
NCT05979792	CD7	R/R CD7+ peripheral T-cell lymphoma	35	Early Phase 1	Not yet recruiting	CD7 CAR T therapy
NCT05909527	CD7	R/R T-cell lymphoblastic leukemia/lymphoma	30	Phase 1/2	Active, not recruiting	Anti-CD7 CAR T cells
NCT05907603	CD7	R/R CD7+ hematologic malignancies	8	Early Phase 1	Completed	RD13-02 cell infusion
NCT05902845	CD7	R/R CD7+ hematologic malignancies	4	Early Phase 1	Completed	RD13-02 cell infusion
NCT05827835	CD7	R/R CD7+ malignant hematologic diseases	30	Phase 2	Unknown status	CD7 CAR T cells + allo-HSCT
NCT05716113	CD7	R/R CD7+ T-ALL/lymphoblastic lymphoma	20	Early Phase 1	Completed	RD13-02 cell infusion

T-LBL, T-lymphoblastic lymphoma; PTCL, peripheral T-cell lymphoma; CTCL, cutaneous T-cell lymphoma; R/R ETP, relapsed/refractory early T-cell precursor acute lymphoblastic leukemia.

#### CAR T cells anti-CD19

CD19 is predominantly expressed on B-lineage cells [71, 72], but aberrant expression occurs in selected AML subsets, particularly t(8;21) AML and mixed-phenotype acute leukemia [73–75]. CD19 CAR T-cell therapy may therefore be relevant for a limited, biomarker-defined population rather than unselected AML.

In a phase II study of CD19-positive R/R AML, six of 10 patients achieved CR or CR with incomplete count recovery after point-of-care CD19 CAR T-cell infusion [76]. CRS was generally manageable, and no neurotoxicity was reported. Responses were frequently consolidated with allo-HSCT, indicating that CD19 CAR T cells may serve as remission induction for selected patients.

A prospective phase II trial in relapsed t(8;21) AML reported CR or CR with incomplete recovery in 10 of 11 patients after CD19 CAR T-cell therapy [77]. Most responders subsequently underwent allo-HSCT. Relapses were associated with reduced or lost CD19 expression, confirming antigen escape as a clinically relevant limitation. The restricted prevalence and variable density of CD19 in AML require careful immunophenotypic selection and monitoring.

#### Other novel targets

Several additional AML targets are under investigation. FLT3 is expressed on AML blasts and leukemia stem/progenitor cells, and FLT3-directed CAR T cells show antileukemic activity in preclinical models [35, 78–81]. Clinical translation must account for FLT3 expression on normal progenitors and the risk of hematopoietic toxicity. CD38 is broadly expressed across hematologic malignancies and may be increased by IFN-γ; CD38 CAR T cells have shown preclinical activity and early clinical responses in post-transplant R/R AML [34, 82–85]. Other candidate antigens include CD44v6, FRβ, Lewis Y, CD47, and NKG2D ligands. Each has a distinct balance of prevalence, leukemia stem cell coverage, and expression on normal tissues. Gene editing, dual-target CARs, logic-gated receptors, and pharmacologic modulation of antigen density may improve specificity and reduce escape. Because AML antigen expression is heterogeneous and dynamic, target selection will likely require patient-level immunophenotyping and, in some cases, sequential or combinatorial approaches rather than a universal construct.

### CAR-NK cell therapy

CAR T-cell therapy has achieved breakthrough progress in the treatment of AML; however, both autologous and allogeneic CAR T cells still encounter challenges, including severe CRS and off-target effects. CAR-NK cells share with CAR-T cells the ability to recognize target antigens via their CAR construct and to induce apoptosis through the release of cytotoxic granules containing perforin and granzymes, as well as the secretion of cytokines such as IFN-γ and TNF-α [86, 87]. However, the key distinctions between CAR-NK and CAR-T cells lie in their longevity, capacity for memory formation, and the magnitude and duration of effector functions. Notably, CAR-NK cells exhibit a markedly lower risk of severe CRS and ICANS compared with CAR-T cells, a feature attributable primarily to their highly transient *in vivo* persistence [86]. Furthermore, the lack of GVHD risk in the allogeneic setting renders CAR-NK cells particularly suitable for “off-the-shelf” therapy. Nevertheless, this same transient nature limits their long-term persistence and sustained anti-leukemia activity, presenting a trade-off between safety and durability that must be addressed through optimized manufacturing or combination strategies.

In a phase 1 clinical study [88], Huang *et al*. selected the CD33-targeting domain from CAR T cells and transduced this optimized CAR construct into umbilical cord-derived NK cells using a retroviral vector. Preclinical efficacy and safety studies were conducted *in vitro* and *in vivo*. Ten patients with R/R AML, aged 18–65 years, received one or more infusions of anti-CD33 CAR-NK cells following a conditioning regimen. The results indicated that on Day 28 after reinfusion therapy, six patients achieved MRD-negative CR, suggesting that the efficacy of CD33 CAR-NK cells was comparable to that of CD33 CAR T cells. However, they exhibit limited toxicity toward HSCs. Apart from Bone marrow suppression, no grade 3–4 adverse events were observed, and the suppression resolved within 1 month. No cases of ICANS or GVHD were reported following CAR-NK infusion. Only one patient developed grade 2 CRS with persistent fever. These findings demonstrate the efficacy and safety of CD33 CAR-NK cells in patients with R/R AML.

CD123 CAR-NK cells have shown potent activity against AML in preclinical models. Cord-blood-derived CD123 CAR-NK cells reduced leukemia burden while showing limited toxicity toward normal hematopoietic progenitors in one study [89]. Engineering strategies, such as IL-15 expression, inducible safety switches, and optimization of CAR signaling may improve persistence and control. Clinical trials are needed to determine whether these advantages translate into durable responses.

CAR-NK therapy demonstrates high efficacy and low toxicity in AML, with transformative potential for patients with R/R. Furthermore, CAR-NK cell therapies targeting CD38, CLL-1, FLT3, NKG2D, and other antigens are under active investigation [90–93]. Multitarget combinations are also being attempted with the expectation that this “off-the-shelf” cellular immunotherapy can further enhance antitumor effects while reducing severe adverse reactions, such as CRS and extramedullary disease suppression.

### CAR-macrophage cell therapy

CAR T-cell therapy is highly effective in hematological malignancies but remains limited in solid tumors. Although CAR-NK cells reduce the risks of CRS and ICANS, their activity may be impaired within suppressive tumor microenvironments, where factors, such as TGF-β, IL-4, and IL-10, restrict infiltration and effector function [94]. These limitations have prompted interest in macrophages as alternative CAR-engineered effector cells because of their intrinsic capacity to infiltrate tissues and function within immunosuppressive niches. CAR-Ms use the phagocytic and antigen-presenting functions of macrophages to recognize and engulf antigen-positive tumor cells while recruiting T-cell and NK-cell responses [95, 96]. Their tissue infiltration and ability to produce proinflammatory signals provide a rationale for use in suppressive tumor microenvironments. Evidence in myeloid malignancies remains preclinical. CD26-directed CAR-Ms constructed from murine macrophages preferentially phagocytosed CD26-positive chronic myeloid leukemia cells and delayed disease progression in mouse models [97]. Although this study was conducted in CML rather than AML, it provides proof of concept that CAR-Ms can target myeloid leukemia stem-cell-associated antigens. AML-specific targets and models require further study.

Clinical translation of CAR-Ms is limited by macrophage plasticity, short persistence, restricted proliferation, inefficient ex vivo expansion and gene transfer, and uncertain consistency of manufactured products [27, 96, 98]. Excessive IL-6 or TNF-α release may cause inflammatory toxicity, while the safety of allogeneic CAR-Ms is largely unknown. Future development should improve target selection, vector design, persistence, and resistance to microenvironmental suppression, potentially through CRISPR-based engineering or combination with checkpoint blockade.

### TCR-T therapy

TCR-T cells are genetically modified to express receptors that recognize peptide-HLA complexes [99]. Unlike CARs, TCRs can target intracellular proteins such as WT1, substantially expanding the antigen repertoire [100]. HLA restriction may improve leukemia specificity but limits applicability to patients with compatible alleles. Additional engineering, including costimulatory modules and PD-1/CD28 switch receptors, can enhance proliferation and convert inhibitory PD-L1 signaling into activation [101, 102].

WT1 is a leading TCR-T target because it is highly expressed in AML and has limited expression in most normal tissues. Chapuis *et al*. [103] infused WT1-specific TCR-engineered donor CD8^+^ T cells after transplantation in 12 high-risk patients. At a median follow-up of 44 months, relapse-free survival was 100%, compared with 54% in a concurrent comparison group, supporting prophylactic WT1-directed therapy after HCT.

WT1-directed therapy still carries potential on-target, off-tumor toxicity because low-level expression occurs in renal podocytes, ovarian granulosa cells, and CD34^+^ hematopoietic cells. Antigen downregulation may also permit relapse. Mutated NPM1 provides a more leukemia-specific intracellular target in the 30–35% of AML cases carrying this alteration. An HLA-A*02:01-restricted NPM1-mutant peptide, CLAVEEVSL, was recognized by engineered T cells that lysed NPM1-mutated AML cells and controlled leukemia in a mouse model [104]. These data support further development of mutation-specific TCRs.

TCR-T therapy has demonstrated feasibility and partial clinical benefits in the treatment of AML. Despite the presence of technical bottlenecks, the discovery of novel targets, optimization of T-cell functions, and improvement in manufacturing processes hold promise for making TCR-T therapy a vital pillar in the cellular immunotherapy of AML. Currently, limited antigens, such as WT1, are utilized, and it is crucial to screen for highly specific targets to reduce off-target risks. The next step necessitates the validation of long-term survival benefits through large-scale Phase II/III trials.

## Conclusion

Cellular immunotherapy has produced meaningful responses in heavily pretreated AML, particularly with CAR T cells targeting CD123, CLL-1, CD7, and aberrant CD19. UniCAR and CAR-NK platforms add controllability and off-the-shelf potential. However, AML lacks a universal leukemia-specific surface antigen, and effective targeting often damages normal myelopoiesis. Prolonged cytopenias, infection, CRS, antigen loss, limited persistence, and the suppressive bone marrow microenvironment continue to restrict durability and safety. Progress will require patient-specific antigen selection and strategies that preserve or restore hematopoiesis. Switchable or logic-gated CARs, suicide systems, dual-target constructs, edited effector cells, and rational sequencing with hypomethylating agents, FLT3 inhibitors, or allo-HSCT may improve the therapeutic window. CAR-NK, CAR-M, and TCR-T approaches broaden the available effector cells and antigen classes, but most remain supported by early clinical or preclinical evidence. Future trials should prioritize durable remission, infection-related mortality, hematopoietic recovery, and comparative value over response rate alone. Cellular immunotherapy is likely to become a component of multimodal AML treatment, with its role defined by molecular subtype, antigen profile, disease burden, and transplant eligibility.

## Data Availability

No datasets were generated or analyzed during the current study.
